# The causal relationship between abdominal obesity and lower bone mineral density: A two-sample mendelian randomization study

**DOI:** 10.3389/fgene.2022.970223

**Published:** 2022-10-13

**Authors:** Xiang-xuan Wang, Kai-nan Lin, Wen-chen Xu, Hui Chen

**Affiliations:** ^1^ Department of Pediatric Orthopedics, Fujian Branch of Shanghai Children’s Medical Center Affiliated to Shanghai Jiaotong University School of Medicine, Fuzhou, China; ^2^ Department of Pediatric Orthopedics, Fujian Children’s Hospital, Fuzhou, China; ^3^ Department of Pediatric Orthopedics, Fujian Children’s Hospital, College of Clinical Medicine for Obstetrics and Gynecology and Pediatrics, Fujian Medical University, Fuzhou, China

**Keywords:** abdominal obesity, bone mineral density, with waist circumference, hip circumference, waist-to-hip ratio, mendelian randomization

## Abstract

**Aims:** The purpose of this study was to assess the causal effect of abdominal obesity on bone mineral density by two-sample Mendelian randomization (MR).

**Methods:** Abdominal obesity was chosen as exposure in this study. Single nucleotide polymorphisms, extracted from Genome-wide association analysis (GWAS) data, which are closely associated with waist circumference (WC), hip circumference (HC), and waist-to-hip ratio (WHR) were used as instrumental variables to perform MR studies. Different site bone mineral density, such as total bone mineral density (TBMD) and forearm bone mineral density (FBMD) were chosen as outcomes. Inverse variance weighted (IVW) was used as the primary method to assess this causality.

**Results:** According to the IVW method (β = −0.177; 95% CI = −0.287, −0.067; *p* = 1.52 × 10^–3^), WC had a negative causal relationship with TBMD, besides, with one standard deviation (SD) higher in HC, there was a 0.195 SD decrease in TBMD (95% CI = −0.279, −0.110; *p* = 6.32 × 10^–6^), and with an increase of one SD in HC was related to a decrease of 0.312 SD in FBMD analyzed by the IVW.

**Conclusion:** This study showed that abdominal obesity has a negative effect on bone mineral density.

## 1 Introduction

Obesity is defined as a body mass index (BMI) ≥ 30 kg/m^2^; it is the state in which excess body fat accumulates to a certain extent and adversely affects health (Bosello et al., 2016; Piché et al., 2020). Although there is a good correlation between BMI and whole-body fat content, it does not accurately reflect the distribution of body fat in the whole body; whereas waist circumference (WC), hip circumference (HC), and waist-to-hip ratio (WHR) can measure the extent of fat accumulation in the abdomen (Fang et al., 2018). The fat distribution in the eastern population is different from that of the western population, which is characterized by abdominal obesity (Lao et al., 2015). Among individuals having the same BMI, it has been shown that the fat content of those with abdominal obesity is higher than that of those with general obesity (Smith, 2015; Lukács et al., 2019; Jayawardena et al., 2021); hence, the influence of abdominal obesity on the metabolic level cannot be ignored. On the one hand, several studies have shown that obesity has a profound effect on the increase in bone mineral density (BMD) and thus, which can play an anti-osteoporosis role (Gkastaris et al., 2020; Qiao et al., 2020; Turcotte et al., 2021); on the other hand, some studies have reached the opposite conclusion suggesting that obesity is mainly characterized by the expansion of adipose tissue and chronic low-level systemic inflammation, this leads to the accumulation of ectopic adipocytes in the bone marrow cavity and may cause a decrease in BMD, which may lead to the development of osteoporosis (Gkastaris et al., 2020; Qiao et al., 2020; Turcotte et al., 2021).

The reasons for contradictions in these studies are as follows: 1) there are several retrospective studies (Qiao et al., 2020); therefore, there may be numerous biases and confounders, and reverse causality cannot be excluded; 2) previous studies did not subdivide obesity into abdominal and general obesity and did not analyze the effects of different obesity types on bone metabolism and BMD separately; and 3) previous studies may have included patients of different races, which may have led to heterogeneity. Therefore, the relationship between obesity, particularly abdominal obesity, and BMD is not yet been fully elucidated. More studies are needed to assess the effects of obesity on BMD to provide rational advice and timely medical interventions for patients with obesity.

**FIGURE 1:**
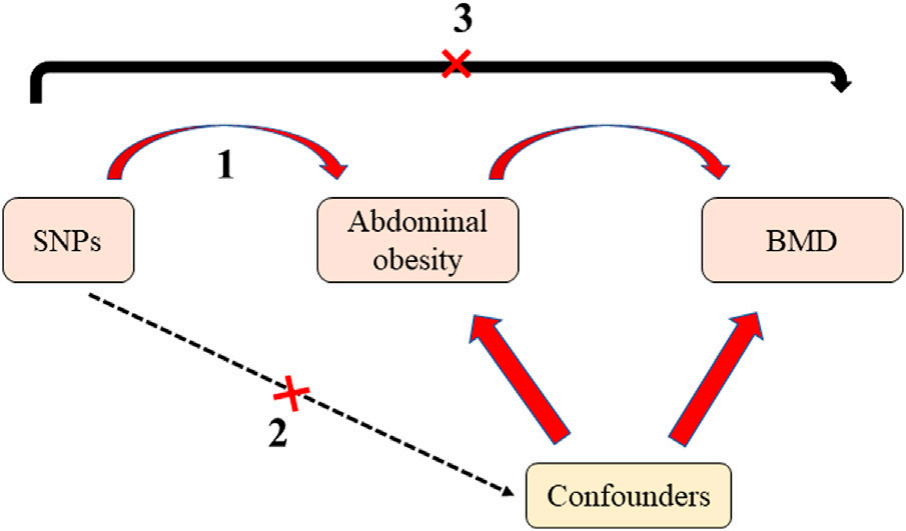
The diagram of Mendelian randomization. There are three assumptions should be met: first, the instrumental variables are closely related to the exposure factor; second, there is no association between the instrumental variables and confounders; third, the instrumental variables should affect the outcome only through exposure factors and not through any other pathway. SNPs: Single nucleotide polymorphisms; BMD: bone mineral density.

Mendelian randomization (MR) uses genetic variation as an instrumental variable to assess whether exposures have a causal effect on outcomes (Davey Smith and Hemani, 2014; Emdin et al., 2017). In contrast to traditional epidemiological studies, the selection of single nucleotide polymorphisms (SNPs) as instrumental variables in MR prevents the relationship between the exposure and outcomes from being influenced by confounding factors, such as environmental factors. As instrumental variables, SNPs are randomly assigned to individuals with gametes, which is similar to a randomized controlled trial and precedes the onset of disease; this is chronological and avoids the effects of reverse causality (Sekula et al., 2016; Birney, 2022). Instrumental variables must meet three important assumptions (Figure 1): first, the instrumental variables are closely related to the exposure factor; second, there is no association between the instrumental variables and confounders; third, the instrumental variables should affect the outcome only through exposure factors and not through any other pathway. Therefore, MR is increasingly used in epidemiology to study etiology.

This study aimed to investigate the relationship between abdominal obesity and BMD using MR studies to provide scientific and reasonable guidance for the obese population.

## 2 Materials and methods

### 2.1 Study design and selection of instrumental variables

In the present study, abdominal obesity-related indicators (including WC, HC, and WHR) were used as exposure factors, while BMD at different sites was used as the outcome for MR analysis. First, we selected SNPs that were strongly associated with exposure (based on *p* < 5.0 × 10^–8^), as instrumental variables from the Genome-wide association analysis (GWAS) data published by Shungin et al. (2015), which was a genome-wide association meta-analysis of WC and HC-related traits in up to 224,459 individuals. Second, we used *r* ^*2*^ < 0.001/clumping and distance >5000 kb as thresholds to remove genes with linkage disequilibrium. Next, we used the Phenoscanner (http://www.phenoscanner.medschl.cam.ac.uk) to remove obesity-related SNPs which were associated with the presence of confounding factors (corticosteroid use, estrogen, malnutrition, menopause, coffee or alcohol intake).

GWAS data for whole-body BMD were obtained from public data published by Medina-Gomez et al. (2018). This study included 30 epidemiological studies with approximately 66,628 European individuals. GWAS data for BMD at individual sites (including the forearm, lumbar spine, and femoral neck BMD)were obtained from public data published by Zheng et al. (2015), which identified novel non-coding genetic variants with large effects on BMD (*n* = 53,236) and fracture (*n* = 508,253) in individuals of European ancestry from the general population. To further exclude effects due to weak instrumental variables, we used allele frequency information to reconcile the data and ensure that the effect alleles of IVs in exposure and outcome corresponded to the same alleles. To further exclude bias due to weak instrumental variables, we used *F*-statistics to assess the presence of weak instrumental variables. *F*-statistics (*F*-statistics = β^2^/se^2^)is an index to evaluate the weakness of IVs, since IVW could be easily affected by weak IVs, so we removed weak IVs, namely, SNPs by *F*-statistics ≤ 10 based on previous studies (Zhou et al., 2019; Nazarzadeh et al., 2020; Jin et al., 2022).

### 2.2 Statistical analysis

**FIGURE 2:**
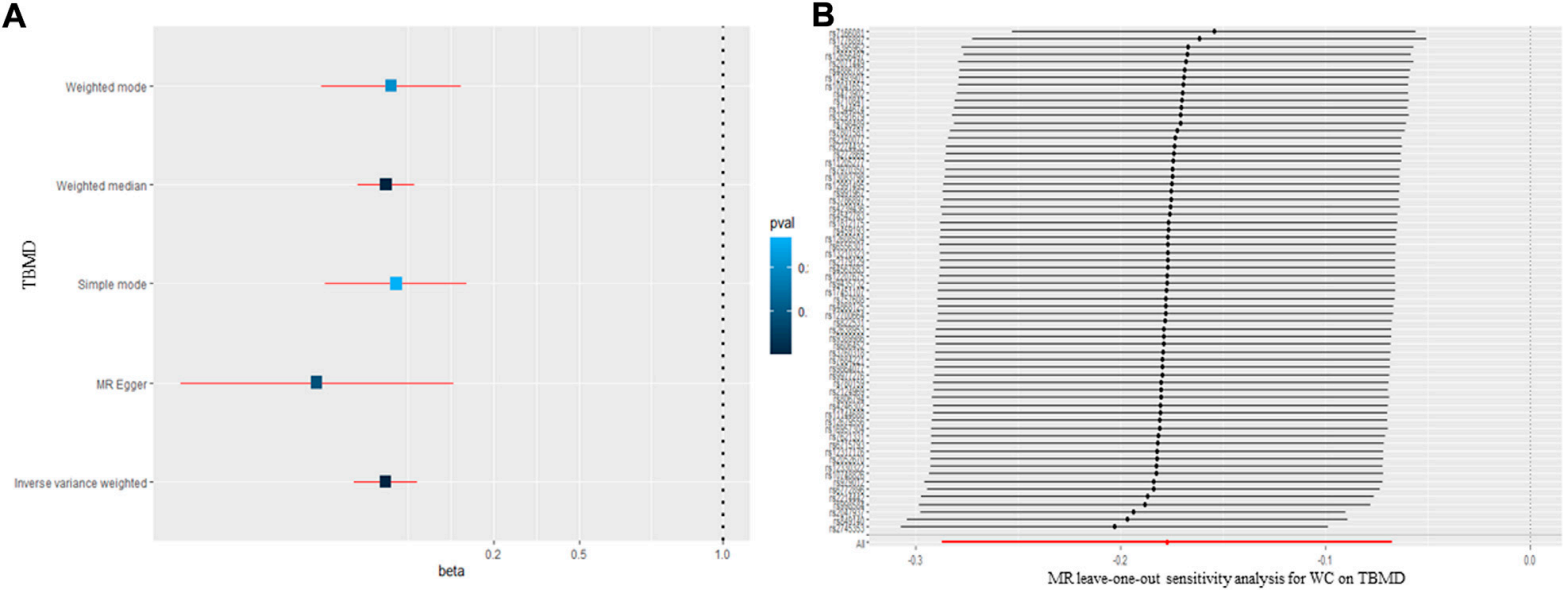
The causal effect of waist circumference on body mineral density **(A)** and **(B)** leave-one-out sensitivity analysis. TBMD: total bone mineral density; MR: Mendelian randomization; WC: waist circumference.

The SNPs extracted were used for MR analysis, and inverse variance weighted (IVW) was used mainly for causality analysis. Additionally, several methods were used to assess causality, including MR-Egger, weighted median, simple mode, and weighted mode. The inverse-variance weighted (IVW) estimator is one of the most popular MR methods that has been widely used in health studies. It has a simple and explicit expression, which combines the estimated causal effects from multiple IVs into a weighted average with the idea borrowed from the fixed-effect meta-analysis literature. The weighted median method uses pooled data and is more tolerant of multivariate genetic variation, even though nearly half of the instrumental variables are unreliable. The MR-Egger regression method, similar to IVW regression, uses data on the effects of genetic variation on disease and exposure factors. In traditional IVW regression, the intercept term in linear regression is forced to be zero, whereas the MR Egger regression estimates the intercept term in the regression equation. A statistically significant non-zero intercept term indicates the presence of directional bias in the selected genetic variants (assuming that pleiotropic effects change the effect in one direction) (Figure 2A) (Jin et al., 2022; Xu et al., 2022). In most cases, IVW was used to assess causal effects. Generally speaking, according to previous studies, when there was absence of heterogeneity and pleiotropy, IVW was the most suitable method for MR analysis, while MR-Egger was recommended when there was the absence of heterogeneity and presence of pleiotropy (Nazarzadeh et al., 2020; Jin et al., 2022).

### 2.3 Sensitivity analysis

Thereafter, to assess the stability and reliability of the MR results, a sensitivity analysis was performed, which included a heterogeneity test using Cochran’s Q test and I^2^ statistics, the inconsistency index (I^2^) statistic, which ranges from 0% to 100% and is defined as the percentage of the observed between-study variability that is due to heterogeneity rather than chance. In this meta-analysis, I^2^ > 50% is designated as a threshold to indicate significant heterogeneity (Higgins et al., 2003). An MR-Egger regression intercept to determine the presence of horizontal pleiotropy, i.e., whether the instrumental variable SNPs, which were strongly correlated with exposure, influenced BMD through other biological pathways. Finally, to test the robustness of the MR results, a leave-one-out test was performed to determine whether the MR results were significantly affected by individual SNP loci.

All data analyses were performed in R software (version 3.6.1) using the “Two sample MR (version 0.5.6)” R package. The Benjamin–Hochberg method was used for the *P*-adjusted value.

## 3 Results

### 3.1 The information of included SNPs

We selected WC, HC, and WHR as exposures, total BMD (TBMD), forearm BMD (FBMD), lumbar spine bone mineral density (LSBMD) and femoral neck bone mineral density (FNBMD) as outcomes to conduct this MR analysis. The SNPs chosen for this study are listed in Supplementary file 1–3. A total of 66 SNPs with a mean of F = 47.22 related to WC (Supplementary Data Sheet S1), 77 SNPs with a mean of F = 54.822 related to HC (Supplementary Data Sheet S2), and 29 SNPs with a mean of F = 48.423 related to WHR were enrolled in this study (Supplementary Data Sheet S3).

### 3.2 Causal relationships of waist circumference on body mineral density

The MR results for the causal effect of WC on BMD are presented in Table 1 and Figure 2A. According to the IVW method (β = −0.177; 95% CI = −0.287, -0.067; *p* = 1.52 × 10^–3^), WC had a negative causal relationship with TBMD. The result of the weighted median method (β = −0.175; 95% CI = −0.276, −0.075; *p* = 6.13 × 10^–4^) was consistent with that of the IVW method. No heterogeneity (Q = 53.30, *p* = 0.709; I^2^ = 33.2 6%) or pleiotropy (intercept = −0.006, *p* = 0.200) was observed in the results by Cochran’s Q, I^2^, and MR-Egger regression. Additionally, the “leave-one-out” analysis indicated that none of the SNPs would affect the MR results (Figure 2B), meaning that the results were reliable and stable. However, as for the relationship between WC and FBMD, all methods showed a null causal effect of WC on FBMD (IVW: β = −0.177; 95% CI = −0.287, −0.067; *p* = 1.52 × 10^–3^; Weighted median: β = −0.177; 95% CI = −0.287, −0.067; *p* = 1.52 × 10^–3^; MR-Egger: β = −0.177; 95% CI = −0.287, −0.067; *p* = 1.52 × 10^–3^) (Supplementary Data Sheet S4). All methods showed a null causal effect of WC on LSBMD and FNBMD as well (Supplementary Data Sheet S4).

**TABLE 1:** Summary of Mendelian randomization results.

Exposure	Outcome	SNP	Method	β	95%CI	Se	*p-*value
WC	TBMD	66	MR Egger	−0.417	−0.893,0.059	0.243	0.091
	TBMD	66	Weighted median	−0.175	−0.276, −0.075	0.051	6.13 × 10^–4^
	TBMD	66	Inverse variance weighted	−0.177	−0.287, −0.067	0.056	1.52 × 10^–3^
	TBMD	66	Simple mode	−0.139	−0.389,0.107	0.126	0.272
	TBMD	66	Weighted mode	−0.157	−0.399,0.086	0.124	0.211
HC	TBMD	77	MR Egger	−0.206	−0.534,0.124	0.168	0.225
	TBMD	77	Weighted median	−0.140	−0.217, −0.0628	0.039	3.83 × 10^–4^
	TBMD	77	Inverse variance weighted	−0.195	−0.279, −0.110	0.043	6.32 × 10^–6^
	TBMD	77	Simple mode	−0.114	−0.290, 0.023	0.090	0.210
HC	TBMD	77	Weighted mode	−0.131	−0.268, 0.006	0.070	0.066
	FBMD	49	MR Egger	−0.524	−1.361, 0.313	0.427	0.226
	FBMD	49	Weighted median	−0.309	−0.578, −0.039	0.137	0.024
	FBMD	49	Inverse variance weighted	−0.312	−0.512, −0.112	0.102	0.0021
	FBMD	49	Simple mode	−0.0154	−0.626, 0.595	0.312	0.961
	FBMD	49	Weighted mode	−0.101	−0.568, 0.367	0.238	0.675

WC: Waist circumference; HC: Hip circumference; TBMD: Total body bone mineral density; FBMD: Forearm bone mineral density; CI: credible interval.

### 3.3 Causal relationships of hip circumference on body mineral density

**FIGURE 3:**
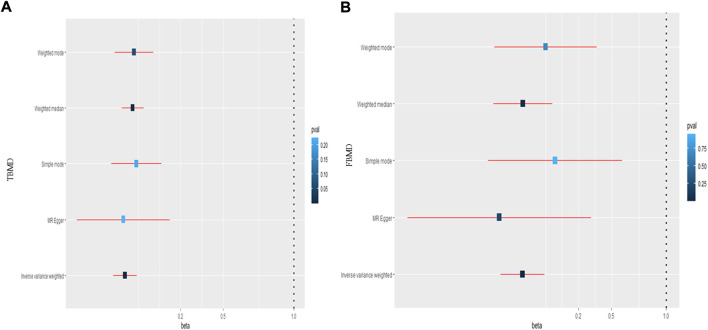
The causal effect of hip circumference on total body mineral density **(A)** and femoral body mineral density **(B)**. TBMD: total bone mineral density; FBMD: femoral body mineral density.

**FIGURE 4:**
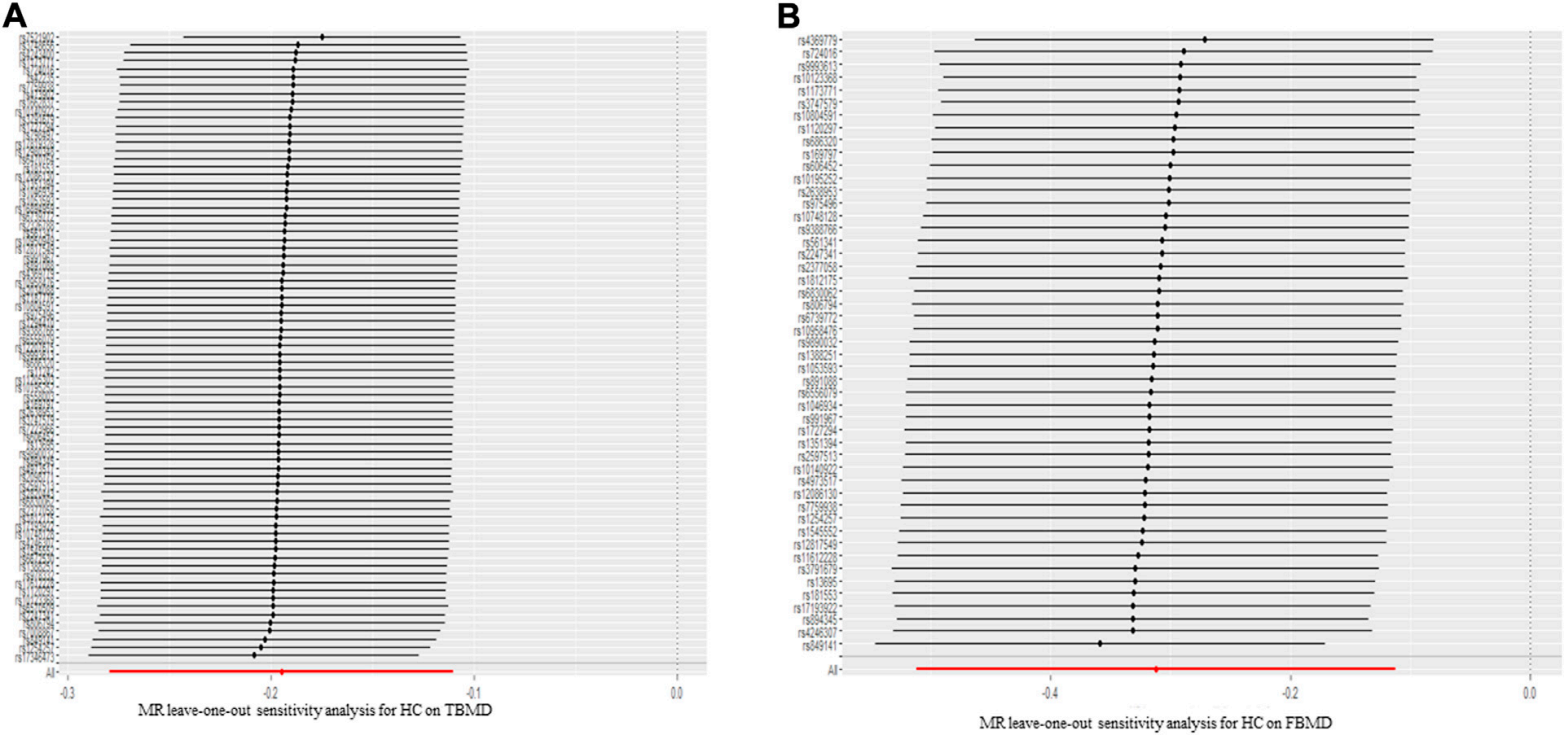
The leave-one-out sensitivity analysis for hip circumference on total body mineral density **(A)** and femoral body mineral density **(B)**. TBMD: total bone mineral density; MR: Mendelian randomization; FBMD: femoral body mineral density.

Regarding the causal effect of HC on BMD, the MR results in Table 1 indicate a negative causal effect between them. With one standard deviation (SD) higher in HC, there was a 0.195 SD decrease in TBMD (95% CI = −0.279, −0.110; *p* = 6.32 × 10^–6^) using the IVW method (Figure 3A). The weighted median (β = −0.140; 95% CI = −0.217, −0.0628; *p* = 3.83 × 10^–4^) showed MR results similar to those of the IVW method. However, heterogeneity (Q = 123.30, *p* = 1.02 × 10^–10^; I^2^ = 60.32%) existed in the MR results between HC and TBMD according to Cochran’s Q and I^2^ statistics. No pleiotropy was found in the causal relationship (intercept = −0.003, *p* = 0.168). Additionally, the results were robust after performing “leave-one-out” analysis (Figure 4A). About the relationship between HC and FBMD, an increase of one SD in HC was related to a decrease of 0.312 SD in FBMD by the IVW method (95% CI = −0.512, −0.112; *p* = 0.0021) (Figure 3B). The weighted median method (β = −0.309; 95% CI = −0.578, −0.039; *p* = 0.024) also detected a negative causal effect of HC on the FBMD. There was heterogeneity (Q = 185.30, *p* = 3.63 × 10^–13^; I^2^ = 75.96%); however, no pleiotropy (intercept = −0.0037, *p* = 0.068) was found in the HC-FBMD causal relationship. The “leave-one-out” analysis indicated that the MR result was stable and reliable. However, all methods showed there were null causal effects of HC on LSBMD and FNBMD (Supplementary Data Sheet S4).

### 3.4 Causal relationships of waist-to-hip ratio on body mineral density

This study also conducted an MR analysis between WHR and BMD. The sample provided sufficient statistical power for the causal analysis of WHR on TBMD; however, no causal relationship was found between exposures and outcomes according to the IVW (β = −0.140; 95% CI = −0.217, −0.0628; *p* = 3.83 × 10^–4^), weighted median (β = −0.140; 95% CI = −0.217, −0.0628; *p* = 3.83 × 10^–4^), and MR-Egger regression methods (β = −0.140; 95% CI = −0.217, −0.0628; *p* = 3.83 × 10^–4^). No heterogeneity (Q = 12.37, *p* = 0.369; I^2^ = 43.26%) or pleiotropy (intercept = −0.0003, *p* = 0.503) was observed in the MR results by Cochran’s Q, I^2^ statistics, and MR-Egger regression. None of the SNPs strongly influenced the overall effect of WHR on TBMD in the leave-one-out analysis. As for the association of WHR on FBMD, the IVW method (β = −0.177; 95% CI = −0.287, −0.067; *p* = 1.52 × 10^–3^) showed a null causal effect between them (Supplementary Data Sheet S4). The weighted median (β = −0.177; 95% CI = −0.287, −0.067; *p* = 1.52 × 10^–3^), MR-Egger method (β = −0.177; 95% CI = −0.287, −0.067; *p* = 1.52 × 10^–3^), and other MR methods were similar to the IVW method. Moreover, none of the MR methods showed there were causal effects of WHR on LSBMD and FNBMD (Supplementary Data Sheet S4).

## 4 Discussion

The current study aimed to detect the causal relationships between abdominal obesity (WC, HC, WHR) and BMD (TBMD and FBMD). To the best of our knowledge, this is the first study to assess the causal relationship between abdominal obesity and BMD in different positions. The results showed that WC had a negative causal effect on TBMD, and HC had negative causal relationships for TBMD and FBMD; this could aid in elucidating the underlying mechanism of osteoporosis and developing treatment strategies for patients with abdominal obesity.

In recent years, the relationship between abdominal obesity and osteoporosis has attracted many scholars due to a large number of individuals in developed societies with obesity and low BMD and the controversial conclusions regarding their association. Laurent et al. found that hip and lumbar spine BMD were higher in the obese population (Maïmoun et al., 2020). A cross-sectional analysis of 3674 participants found that adiposity was positively associated with BMD in premenopausal women; however, it was negatively associated with BMD in postmenopausal women (Bland et al., 2022). Conversely, a large cohort study involving 5268 American participants revealed that abdominal adiposity was negatively associated with BMD, which contradicted the notion that excess fat mass is protective of bone health (Krishnan et al., 2017). Similarly, Kim et al. (2019) concluded that abdominal obesity is related to lower BMD at non-weight-bearing sites in Korean men. Additionally, another study showed that BMD was 0.5 SD lower in the obesity group than in the control group (Kindler et al., 2020).

Various studies have used WC, HC, and WHR traits to determine causal relationships between adiposity and BMD. As for WC, Joseph et al. revealed that it had a negative association with TBMD (Kindler et al., 2019). Additionally, other studies involving 5084 and 4663 participants found that WC was negatively related to BMD(Chen et al., 2019; Hua et al., 2021). These findings are consistent with our results, which showed that WC was negatively associated with TBMD. Moreover, an analysis of 4445 Iranian participants (1900 men and 2545 women) revealed that BMI was related to BMD, while HC or WHR was not (Aghaei Meybodi et al., 2011). Yang et al. (2011) concluded that age and weight, but not HC, contributed the most to the variance in BMD. The discrepancy between these studies and ours might be attributed to the diverse races and different BMD sites. As for the WHR, negative associations were observed between WHR and spine BMD. In addition, a study concerning 1694 Korean women concluded that WHR was negatively associated with spine BMD (Deng et al., 2021). However, there were many limitations to these observational studies due to the existence of confounding factors and reverse causal relationships.

Although many studies have clarified that obesity is related to BMD, the underlying mechanisms have not been well elucidated. Mechanical loading and metabolic factors are the two main factors associated with obesity and BMD. As for mechanical loading, it was found that repeated mechanical loading could increase BMD (Watson et al., 2019; Jeon et al., 2021); however, it might not suffice in obesity. In individuals who are obese, low-grade systemic inflammation can cause an increase in the production of bone marrow adipogenesis, potentially leading to the loss of BMD (Gkastaris et al., 2020). Additionally, leptin, adiponectin, resistin, TNF-α, IL-6, and other metabolic substances are related to the relationship between abdominal obesity and BMD. Leptin has positive or negative effects on BMD depending on the direct or indirect effects exerted by the central-hypothalamic and peripheral pathways; the negative effect seemed to prevail over the positive effect (Rinonapoli et al., 2021). Adiponectin is an adipokine proven to stimulate osteoblastic proliferation by enhancing the expression of cyclooxygenase-2 and bone morphogenetic protein-2 (Pu et al., 2017). Amy et al. revealed that lower levels of adiponectin were found in women who were obese than in women with a normal BMI (Kelly et al., 2020), proving that obesity could be negatively associated with BMD. Moreover, a high level of resistin was found in people who were obese. Although the effects of resistin on BMD are complex, it can increase the proliferation of both osteoblasts and osteoclasts (Kollari et al., 2022). High levels of inflammatory factors, such as TNF-α and IL-6, are found in people who are obese and can induce osteoclast genesis and bone resorption (Aguirre et al., 2014; Zhang et al., 2015). All in all, the potential associations between abdominal obesity and BMD are complex and need additional research to obtain clarity.

Compared with observational studies, this MR analysis has many strengths when investigating the causal relationship between abdominal obesity and BMD. First, the large sample size and the availability of individual-level data provided this study sufficient power to explore the causal effects of abdominal obesity on BMD with high precision. Second, this study minimized unmeasured confounders and bias in observational studies. As for the null causal effects for lumbar spine and femoral neck, the reasons might be attributed to: 1) age heterogeneity; the datasets used in this study are the meta-analysis of many GWAS studies, which including people from different ages groups, the causal effect of obesity on BMD might be neutralized by effects of age, the age-specific MR should be performed to explore the causal effect of obesity on BMD more comprehensively in the following studies; 2) insufficient SNPs; both datasets were published years ago, more people should be included in GWAS studies to identify more obesity and BMD related SNPs, for a more conclusive MR analysis results. Moreover, the current study had some limitations. First, it was difficult to perform further subgroup analyses for BMD based on the original GWAS data. Additionally, whether similar results could be obtained from other races in this analysis was based on the European ancestor.

## 5 Conclusion

In conclusion, this study conducted an MR analysis to detect the causal relationship between abdominal obesity and BMD. It showed that WC was negatively related to TBMD, and HC was negatively related to TBMD and FBMD; this could aid in exploring the potential mechanisms of abnormal BMD levels in patients with abdominal obesity.

## Data Availability

The original contributions presented in the study are included in the article/supplementary files, further inquiries can be directed to the corresponding author.
