# Supplementary material for: The causal relationship between abdominal obesity and lower bone mineral density: A two-sample mendelian randomization study
Source: Front Genet. 2022 Oct 13;13:970223. doi: 10.3389/fgene.2022.970223 (PMC9606644; doi:10.3389/fgene.2022.970223)
Supplement: Supplementary file 2 [file Table1.DOCX]

**Table 1 Summary of Mendelian randomization results**

| Exposure | Outcome | SNP | Method | β | 95%CI | Se | *P-*value |
| --- | --- | --- | --- | --- | --- | --- | --- |
| WC | TBMD | 66 | MR Egger | -0.417 | -0.893,0.059 | 0.243 | 0.091 |
|  | TBMD | 66 | Weighted median | -0.175 | -0.276, -0.075 | 0.051 | 6.13×10^-4^ |
|  | TBMD | 66 | Inverse variance weighted | -0.177 | -0.287, -0.067 | 0.056 | 1.52×10^-3^ |
|  | TBMD | 66 | Simple mode | -0.139 | -0.389,0.107 | 0.126 | 0.272 |
|  | TBMD | 66 | Weighted mode | -0.157 | -0.399,0.086 | 0.124 | 0.211 |
| HC | TBMD | 77 | MR Egger | -0.206 | -0.534,0.124 | 0.168 | 0.225 |
|  | TBMD | 77 | Weighted median | -0.140 | -0.217, -0.0628 | 0.039 | 3.83×10^-4^ |
|  | TBMD | 77 | Inverse variance weighted | -0.195 | -0.279, -0.110 | 0.043 | 6.32×10^-6^ |
|  | TBMD | 77 | Simple mode | -0.114 | -0.290, 0.023 | 0.090 | 0.210 |
| HC | TBMD | 77 | Weighted mode | -0.131 | -0.268, 0.006 | 0.070 | 0.066 |
|  | FBMD | 49 | MR Egger | -0.524 | -1.361, 0.313 | 0.427 | 0.226 |
|  | FBMD | 49 | Weighted median | -0.309 | -0.578, -0.039 | 0.137 | 0.024 |
|  | FBMD | 49 | Inverse variance weighted | -0.312 | -0.512, -0.112 | 0.102 | 0.0021 |
|  | FBMD | 49 | Simple mode | -0.0154 | -0.626, 0.595 | 0.312 | 0.961 |
|  | FBMD | 49 | Weighted mode | -0.101 | -0.568, 0.367 | 0.238 | 0.675 |

WC: Waist circumference; HC: Hip circumference; TBMD: Total body bone mineral density; FBMD: Forearm bone mineral density; CI: credible interval.
