# Supplementary material for: The causal relationship between abdominal obesity and lower bone mineral density: A two-sample mendelian randomization study
Source: Front Genet. 2022 Oct 13;13:970223. doi: 10.3389/fgene.2022.970223 (PMC9606644; doi:10.3389/fgene.2022.970223)
Supplement: Supplementary file 3 [file Table2.DOCX]

**Table S1 Summary of Mendelian randomization results**

| Exposure | Outcome | SNP | Method | β | Se | *P-*value |
| --- | --- | --- | --- | --- | --- | --- |
| WC | FBMD | 47 | MR Egger | 0.074 | 0.536 | 0.891 |
|  | FBMD | 47 | Weighted median | -0.042 | 0.154 | 0.788 |
|  | FBMD | 47 | Inverse variance weighted | -0.273 | 0.128 | 0.032 |
|  | FBMD | 47 | Simple mode | 0.113 | 0.382 | 0.769 |
|  | FBMD | 47 | Weighted mode | 0.124 | 0.346 | 0.721 |
|  | LSBMD | 41 | MR Egger | -0.145 | 0.244 | 0.556 |
|  | LSBMD | 41 | Weighted median | 0.058 | 0.088 | 0.515 |
|  | LSBMD | 41 | Inverse variance weighted | 0.104 | 0.060 | 0.086 |
|  | LSBMD | 41 | Simple mode | -0.012 | 0.193 | 0.952 |
|  | LSBMD | 41 | Weighted mode | -0.012 | 0.191 | 0.951 |
|  | FNBMD | 41 | MR Egger | -0.326 | 0.349 | 0.356 |
|  | FNBMD | 41 | Weighted median | -0.099 | 0.085 | 0.247 |
|  | FNBMD | 41 | Inverse variance weighted | -0.0007 | 0.086 | 0.994 |
|  | FNBMD | 41 | Simple mode | -0.320 | 0.213 | 0.139 |
|  | FNBMD | 41 | Weighted mode | -0.320 | 0.175 | 0.075 |
| HC | LSBMD | 35 | MR Egger | 0.177 | 0.271 | 0.518 |
|  | LSBMD | 35 | Weighted median | 0.063 | 0.085 | 0.456 |
|  | LSBMD | 35 | Inverse variance weighted | 0.080 | 0.065 | 0.219 |
|  | LSBMD | 35 | Simple mode | 0.162 | 0.162 | 0.909 |
|  | LSBMD | 35 | Weighted mode | 0.133 | 0.133 | 0.649 |
|  | FNBMD | 35 | MR Egger | -0.244 | 0.263 | 0.361 |
|  | FNBMD | 35 | Weighted median | -0.126 | 0.073 | 0.085 |
|  | FNBMD | 35 | Inverse variance weighted | -0.044 | 0.064 | 0.485 |
|  | FNBMD | 35 | Simple mode | -0.139 | 0.144 | 0.343 |
|  | FNBMD | 35 | Weighted mode | -0.151 | 0.108 | 0.174 |
| WHR | TBMD | 28 | MR Egger | 0.153 | 0.277 | 0.583 |
|  | TBMD | 28 | Weighted median | -0.220 | 0.071 | 0.002 |
|  | TBMD | 28 | Inverse variance weighted | -0.121 | 0.061 | 0.046 |
|  | TBMD | 28 | Simple mode | -0.249 | 0.147 | 0.101 |
|  | TBMD | 28 | Weighted mode | -0.236 | 0.124 | 0.067 |
|  | FBMD | 18 | MR Egger | 0.748 | 0.888 | 0.412 |
|  | FBMD | 18 | Weighted median | 0.152 | 0.237 | 0.521 |
|  | FBMD | 18 | Inverse variance weighted | -0.172 | 0.211 | 0.415 |
|  | FBMD | 18 | Simple mode | 0.173 | 0.473 | 0.719 |
|  | FBMD | 18 | Weighted mode | 0.340 | 0.340 | 0.332 |
|  | LSBMD | 16 | MR Egger | 0.732 | 0.378 | 0.073 |
|  | LSBMD | 16 | Weighted median | 0.165 | 0.134 | 0.219 |
|  | LSBMD | 16 | Inverse variance weighted | 0.074 | 0.092 | 0.421 |
|  | LSBMD | 16 | Simple mode | 0.206 | 0.193 | 0.304 |
|  | LSBMD | 16 | Weighted mode | 0.214 | 0.158 | 0.195 |
|  | FNBMD | 16 | MR Egger | -0.297 | 0.489 | 0.554 |
|  | FNBMD | 16 | Weighted median | -0.032 | 0.123 | 0.796 |
|  | FNBMD | 16 | Inverse variance weighted | -0.044 | 0.114 | 0.700 |
|  | FNBMD | 16 | Simple mode | -0.034 | 0.235 | 0.887 |
|  | FNBMD | 16 | Weighted mode | -0.113 | 0.190 | 0.560 |

WC: Waist circumference; HC: Hip circumference; TBMD: Total body bone mineral density; FBMD: Forearm bone mineral density; LSBMD: lumbar spine bone mineral density; FNBMD: femoral neck bone mineral density; WHR: waist-to-hip ration; credible interval.
